# Feasibility and Usability of a Web-Based Peer Support Network for Care Partners of People With Serious Illness (ConnectShareCare): Observational Study

**DOI:** 10.2196/70206

**Published:** 2025-06-11

**Authors:** Aricca D Van Citters, Megan M Holthoff, Colleen Young, Sarah M Eck, Amelia M Cullinan, Stephanie Carney, Elizabeth A O'Donnell, Joel R King, Malavika Govindan, David Gustafson, Stephanie C Tomlin, Anne B Holmes, Ann D Bradley, Brant J Oliver, Matthew M Wilson, Eugene C Nelson, Amber E Barnato, Kathryn B Kirkland

**Affiliations:** 1 Dartmouth Institute for Health Policy and Clinical Practice, One Medical Center Drive, WTRB Level 5, Lebanon, NH, 03756, United States, 1 6036465668; 2 Mayo Clinic Connect, Mayo Clinic, Rochester, MN, United States; 3 Scientific Health, Lyme, NH, United States; 4 Palliative Medicine, Geisel School of Medicine at Dartmouth, Lebanon, NH, United States; 5 Section of Palliative Medicine, Dartmouth Health, Lebanon, NH, United States; 6 Dartmouth Health, Lebanon, NH, United States; 7 Alice Peck Day Memorial Hospital, Lebanon, NH, United States; 8 Patient and Family Advisor, United States; 9 College of Engineering, University of Wisconsin - Madison, Madison, WI, United States; 10 Departments of Community & Family Medicine, Psychiatry, Geisel School of Medicine at Dartmouth, Lebanon, NH, United States; 11 Office of Care Experience, The Value Institute, Dartmouth Health, Lebanon, NH, United States

**Keywords:** serious illness, care partner, caregivers, peer-support, self-help groups, social networking, internet, peer-to-peer

## Abstract

**Background:**

While it can be rewarding to provide care for a person with serious illness, care partners are often unprepared to manage and cope with the physical and emotional stresses that arise with disease progression and bereavement.

**Objective:**

We aimed to evaluate membership enrollment, engagement, and experiences within a web-based peer support network for active and bereaved care partners of people with serious illness.

**Methods:**

We conducted a formative evaluation of the ConnectShareCare peer-to-peer web-based support network, which targeted care partners of people with serious illness residing in the northeastern United States. Recruitment methods included marketing postcards, flyers, listserv messages, and referrals from community stakeholders, peers, and clinicians. Enrollment occurred through a self-guided, web-based process. Study participants included members enrolled in ConnectShareCare between April 2021 and June 2023. We used the network’s analytics dashboard (eg, registration, usage, and notification logs) to evaluate membership enrollment and engagement in discussions. We used surveys of a subset of members to assess experiences, including satisfaction, ability to find meaning by supporting others, and value and opportunities for improvement.

**Results:**

Over 2 years, the network enrolled 250 members, with an average of 9 new members per month. Among 193 members providing information, most (58%, n=112) identified as active care partners, 17% (n=33) identified as bereaved care partners, and 27% (n=52) chose not to specify their role. Two-thirds of the 250 members did not post, 20% (n=50) posted 1‐10 times, 6% (n=14) posted 11‐25 times, 6% (n=15) posted 26-100 times, and 3% (n=7) posted more than 100 times. On average, 19 members posted per month resulting in 166 member posts per month. Moderators (1 community manager, 2 volunteer mentors, and 2 project team members) supported members with an average of 111 posts/month. In total, 187 discussion topics were created, including 42% (n=78) started by members and 58% (n=109) started by moderators. Seventy-eight discussion topics had 10 or more posts associated with them. The most frequent discussion topics focused on “check-ins” and “sources of joy and hope.” Among 18 care partner members who completed a research survey, 69% (11/16) reported connecting with at least 1 person and 62% (10/16) reported that ConnectShareCare helped them find meaning and purpose by supporting others. Most reported satisfaction with support (12/16, 75%) and information (14/16, 88%) through the network. Although most noted that ConnectShareCare was easy to use (10/17, 59%), respondents were less likely to easily find the information they were seeking (6/16, 38%). Survey respondents found value in peer connection and support and identified opportunities to improve navigation of resources and engagement of members.

**Conclusions:**

Care partners of people with serious illness can use a web-based peer support network to find meaningful and useful support and information. Additional work is needed to identify the impact of the network on distress, social connectivity, and support programming.

## Introduction

In the United States, approximately 38 million unpaid care partners (often family members or friends) provided 36 billion hours of care in 2021, with an estimated value of US $600 billion [1]. While caring for a loved one with a serious illness can bring deep meaning and satisfaction, it can also be challenging [2]. Care partners of people with serious illness are often unprepared to manage and cope with the burden of caregiving and the circumstances that arise with disease progression and bereavement. They often have insufficient communication with clinicians; receive inadequate support and educational materials; and encounter burdens, challenges, and unmet needs [3,4]. Most family care partners work full-time, and many have their own health issues [1]. Addressing care partner needs is a pressing health, economic, and social imperative.

There is a growing literature on web-based peer support networks for care partners of people with serious illness, and such networks typically contain a platform to exchange information and provide peer support [5]. Evidence on the impact of web-based peer support networks is promising, with the potential to improve emotional well-being, mood, burden, quality of life, self-efficacy, decision-making, confidence, and relationship quality [6-13]. Building on this evidence base, we co-designed and implemented ConnectShareCare [14], a web-based peer support network to assist active and bereaved care partners of adults with a serious illness in providing each other with emotional support and exchanging helpful information and resources.

Despite the growth in web-based peer support networks for care partners of people with serious illness, there is limited information describing growth in membership enrollment, engagement of members in discussions, and experiences of members participating in such networks. The primary objective of this study is to describe the feasibility and usability of the ConnectShareCare web-based peer support network for active and bereaved care partners.

## Methods

### Study Design

We conducted a formative evaluation to examine the feasibility and usability of ConnectShareCare, as defined by growth in membership enrollment, engagement in discussions, and member experiences using the network. The co-design process supporting the development of the web-based peer support network has been described separately by O’Donnell et al [14].

### Setting

This study was conducted at a large, rural academic medical center in the northeastern United States. While the web-based peer support network targeted care partners of people with serious illness [15] residing in New Hampshire and Vermont, participation was not restricted by geographic region or by receipt of health care within the academic medical center.

### Participants

#### Network Recruitment and Enrollment

Participants included people enrolled in the ConnectShareCare network (“members”) between April 2021 and June 2023. We recruited people to the ConnectShareCare network via multiple methods, including invitations from co-design team members; marketing postcards and flyers posted within the community (eg, community boards, libraries, or senior centers); presentations to community stakeholders (eg, health fairs, networking and learning meetings, or local hospices); and posts to local web-based resources (eg, community listservs or recruitment via peers). We also worked with local health systems to promote referrals from clinicians. We shared referral materials with clinicians and support group providers within the community, health system, and community organizations (eg, hospice care, visiting nursing services, or Veterans Affairs clinics). People enrolled in the network using a self-guided, web-based enrollment process.

#### Recruitment for Survey on Member Experiences

Among the subset of 181 members who enrolled in the network between April 2021 and August 2022, a total of 55% (n=100) agreed during the enrollment process to be contacted for research. We contacted individuals via email between September and November 2022 to complete an anonymous web-based survey to share their experiences using ConnectShareCare. Potential survey participants received reminder emails 7 and 14 days later. We also posted the anonymous survey link on the ConnectShareCare network and invited people who initially opted out of participating in additional research to complete the survey.

### Description of the ConnectShareCare Platform

#### Peer Connection

The ConnectShareCare network provided participants with the ability to connect with each other using “support group” forums, categorized as: active caring, grief and loss, and news and announcements. Discussion topics within these forums were often initiated by moderators, but members also established new discussion topics. The network also featured “private messaging” to facilitate writing to another member directly, and “member stories” (including poetry, music, and artwork), which offered a place to share member experiences and learn from one another.

#### Resources

The network provided members with access to pertinent web-based resource and local and related events and programming. Five categories of resource materials were available, including: planning ahead (eg, state-specific advance directives or care guides); practical resources (eg, housing, insurance, bills, legal, transportation, or food); emotional resources (eg, mental health, finding a therapist, or healing after loss); communication resources (eg, connecting with care providers, interpreters, or finding help); and family resources (eg, managing children, partners, or intimacy). Upcoming events included items such as “Drop-In Mindfulness,” “The Parkinson’s Workshop,” and “Bereavement Hiking Group.”

#### Roles and Responsibilities Within the Network

The network was overseen by a community manager, who set the strategic direction and tactics of the network concerning enrollment and engagement [16]; developed relationships with health care and community partners; recruited, trained, and managed volunteer mentors; and served as the primary moderator of the network. The community manager held a Master of Public Health degree and was supported by an external consultant with significant expertise in community management of web-based peer support networks. Two volunteer mentors were recruited based on their pre-existing skill sets and came to the platform with essential professional and life experiences. Each had formal training in providing complementary health services (eg, Reiki and social work), but were not employed by the health system. In addition to serving as moderators when the community manager was unavailable, volunteer mentors were consistently present on the network, assisting in connecting members, providing resources, and modeling behaviors and posting practices. The network was supported by 2 project team members who were responsible for administrative tasks and served as moderators when the community manager was unavailable.

The moderation structure of the network was modeled after established practices [17]. The community manager signed-in multiple times a week to intentionally nurture and facilitate connection among members with shared experiences; establish new discussion topics, post replies, and ask questions to promote conversation and increase retention of members; and identify or address pressing needs (eg, inappropriate or potentially offensive posts, posts offering unvetted medical advice, etc). When the community manager was unable to fulfill the moderator position (eg, vacation or personal days), volunteer mentors or project team members assumed the moderator role. Community management guidelines (Multimedia Appendix 1) were established and provided to anyone fulfilling the position to clarify expectations and ensure continuity.

#### Safety Monitoring

The community manager, volunteer mentors, and project team members worked together to monitor all posted discussions for potential safety concerns (eg, reference to self-harm or violence) and noncompliance with community guidelines (eg, kindness; digital safety; avoidance of bullying, marketing, or medical advice; etc; Multimedia Appendix 1). To ensure adequate response time, the community manager or designated team member monitored automated safety notifications. A keyword monitoring function was used, which did not prevent posts from being visible, but triggered an email notification to the community manager and project team members when specific keywords were used. Keywords included terms related to self-harm, suicide, and violence (eg, “suicide,” “overdose,” “kill,” “die,” “attack,” or “threaten”). Community members could also report discussions, which generated a similar notification. The reporting feature included an option for a free-text response from the flagger, which provided context for why the discussion was reported. To further bolster security and safety, discussion topics were made viewable only to registered members of the ConnectShareCare community, and new members were restricted from posting hyperlinks during their first 10 days.

### Ethical Considerations

This study was approved by the Dartmouth Health Institutional Review Board (study #02000907). Individuals who enrolled in the network were informed in the “terms of use” that usage data would be collected and used to improve the network. Those who did not agree to the terms of use were unable to participate in the network. The Institutional Review Board granted a waiver of written documentation of consent for the survey component of this study, and the survey introduction included language describing the voluntary nature of this study, confidentiality, and the absence of identifiable data (Multimedia Appendix 2). No identifiable data was collected, and participants were not compensated for their engagement.

### Variables

This study evaluated feasibility via growth in membership enrollment and engagement, and usability via experience of use questions, as defined in Table 1. We used the analytics dashboard provided by the platform vendor, CareHubs, to monitor enrollment and engagement in the network. We used anonymous electronic surveys (Multimedia Appendix 2) to understand usability for a subset of participants. The anonymous survey assessed care partner characteristics, ease of use, value, and opportunities for improvement.

**Table 1.** Feasibility and usability metrics used to assess the ConnectShareCare network.

Metric		Definition	Data source
Feasibility			
	Growth in membership enrollment: new members (cumulative and new enrollments per month)	Count of new members enrolled during the month (excluding duplicate entries, test accounts, and members of the project team).	Network analytics: registration log
	Engagement: total posts per month	Total posts per month by enrolled users (stratified by members and moderators).	Network analytics: usage log
	Engagement: total members posting per month	Number of members posting per month (includes members and moderators).	Network analytics: usage log
Usability			
	Experience: satisfaction (with support received and information found)	Proportion of respondents who report “very satisfied” or “somewhat satisfied” with (1) the support received and (2) the information found through the network.	Anonymous survey
	Experience: ability to find meaning by supporting others	Proportion of respondents who “agree” or “strongly agree” that the network helped them find meaning and purpose by supporting others.	Anonymous survey
	Ease of use of the network	Proportion of respondents who “agree” or “strongly agree” to “the website was easy to use” and “I was able to easily find information I was looking for.”	Anonymous survey
	Perceived value and opportunities for improvement of the network (from free text responses)	Strengths: (1) What do you like most about the ConnectShareCare support network? (2) Please share an example of a time where ConnectShareCare made a positive impact on your day, or on your ability to connect with others, or manage daily activities. Opportunities: What could we do to improve the: (1) support available through ConnectShareCare? (2) information and resources you found through ConnectShareCare?	Anonymous survey

### Statistical Methods

We used statistical process control c Charts [18] to identify changes in enrollment and engagement over time. We used descriptive statistics to summarize categorical information from survey responses. We conducted descriptive statistics with SPSS (version 28; IBM Corp). Subgroup analyses were not conducted due to the small sample size. Missing data were excluded on an analysis-by-analysis basis.

We used thematic analysis to derive qualitative insights into the benefits, limitations, and improvement opportunities identified in free-form text responses to survey questions. Two researchers (JRK and ADVC) independently reviewed all survey responses to familiarize themselves with the data and developed a codebook using inductive methods. Responses were coded by JRK and all coded responses were reviewed by ADVC. JRK and ADVC are experienced qualitative researchers, and JRK has experience in being a care partner for a person with serious illness. Differences in coding decisions were resolved through discussion. To ensure reliability, findings were reviewed with the network moderator and research team. We conducted qualitative analyses using Atlas.ti (version 23, ATLAS.ti Scientific Software Development GmbH) .

## Results

### Participants

#### Characteristics of Network Members

During this study, 250 people enrolled in ConnectShareCare. Among members providing additional information about themselves upon enrollment (n=193), most (n=112, 58%) identified as an active care partner, 17% (n=33) identified as a bereaved care partner, and 27% (n=52) chose not to specify their role. Some individuals identified as both active and bereaved care partners. People most commonly cared for a spouse or partner (n=77, 50%); a child, grandchild, or niece or nephew (n=24, 15%); a parent (n=17, 11%); a sibling (n=8, 5%); a friend or neighbor (n=6, 4%); or another person (n=23, 15%).

#### Characteristics of Survey Respondents

A subset of care partner members (n=18) completed anonymous surveys on their experience with the network, including 13 engaged members (who signed in to the network at least once in the prior 90 d), 1 inactive member (who had not signed in to the network within the prior 90 d), and 4 members responding from an anonymous link provided on the website. At the time of survey completion, 44% (n=8) had been enrolled in ConnectShareCare for 12 or more months; 39% (n=7) for 4‐12 months, and 17% (n=3) for 1‐3 months.

As shown in Table 2, most survey respondents (n=15, 83%) were female, and all spoke English as their primary language. The median age was 68.5 (range 42‐86, IQR 54-74) years. Most (n=15, 83%) had a college degree.

Over half of the survey respondents (n=11, 61%) identified as bereaved. Most respondents cared for their spouse or life partner (n=13, 72%), with a smaller proportion caring for a child or parent. Respondents were care partners for people with cancer (n=7, 39%), dementia (n=4, 22%), Parkinson disease (n=3, 17%), cardiac conditions (n=2, 11%), lung conditions (n=1, 6%), or did not state the type of condition (n=3, 17%).

**Table 2.** Characteristics of care partners responding to an anonymous survey about their experiences with ConnectShareCare (N=18).

	Survey respondents
Gender, n (%)	
Male	3 (17)
Female	15 (83)
Age (years), median (IQR)	68.5 (54-74)
Primary language, n (%)	
English	18 (100)
Education level, n (%)	
High school diploma or equivalent	1 (6)
Some college	2 (11)
College graduate	7 (39)
Master’s or doctoral degree	8 (44)
Care partner type, n (%)	
Bereaved	11 (61)
Active	7 (39)
Relationship of care partner to person with serious illness, n (%)	
Spouse or partner	13 (72)
Child	3 (17)
Parent	1 (6)
Not stated	1 (6)
Length of time as a care partner, n (%)	
Active (<12 months)	1 (6)
Active (12 or more months)	4 (22)
Bereaved (<12 months)	7 (39)
Bereaved (12 or more months)	4 (22)
Not stated	2 (11)
Diagnosis of person with serious illness, n (%)	
Cancer	7 (39)
Dementia	4 (22)
Parkinson disease	3 (17)
Heart disease (eg, stroke)	2 (11)
Lung disease (eg, chronic obstructive pulmonary disease or emphysema)	1 (6)
Not stated	3 (17)

### Main Results

#### Growth in Membership Enrollment

**Figure 1.**
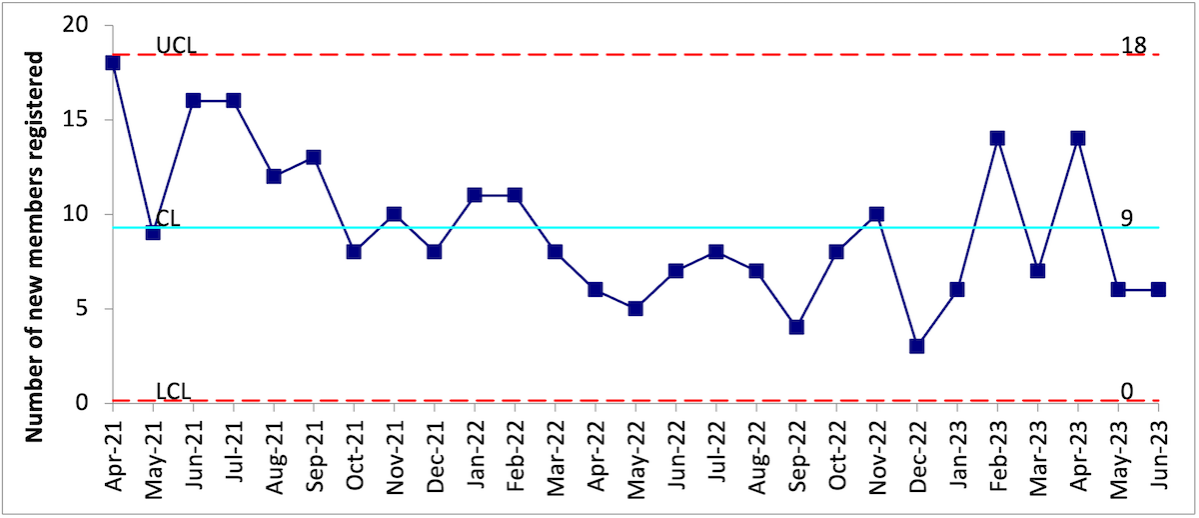
Number of members registered on the ConnectShareCare network each month, between April 2021 and June 2023 (c Chart Statistical Process Control analysis). The mean number of monthly registrations is represented by the turquoise (solid) line. The UCLs and LCLs are represented by the red dash lines. CL: confidence limit; LCL: lower confidence limit; UCL: upper confidence limit.

As shown in Figure 1, a total of 250 members enrolled during this study period, with an average of 9 members enrolling per month. Enrollment of new members was more rapid during the initial testing phase (April 2021 through July 2021), and a lower rate of enrollment was observed between March 2022 and October 2022.

Most participants (n=142, 57%) were referred by a staff or clinician at the health system. Others were referred by: friends, neighbors, or family members (n=34, 14%); community organizations (n=29, 12%); community listservs (n=23, 9%); flyers or pamphlets (n=11, 4%); or another source (n=20, 8%).

#### Engagement: Total Posts Per Month and Members Posting Per Month

**Figure 2.**
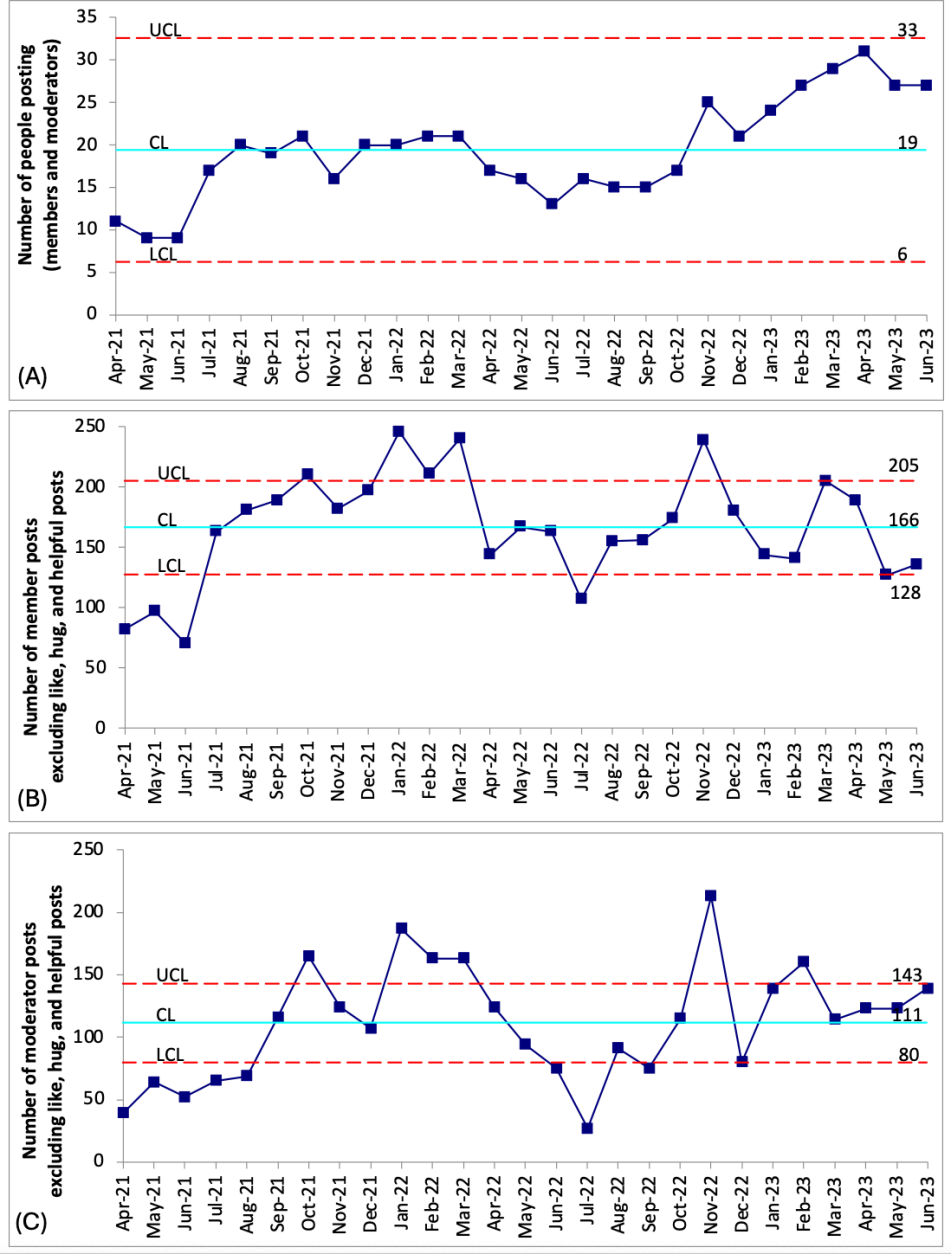
Number of (A) member posts, (B) moderator posts, and (C) total number of individuals (members and moderators) posting each month on the ConnectShareCare network (c Chart Statistical Process Control analysis). The mean is represented by the turquoise (solid) line. The UCLs and LCLs are represented by the red dash lines. CL: confidence limit; LCL: lower confidence limit; UCL: upper confidence limit.

An average of 166 posts were made by members each month (Figure 2A), whereas moderators (1 community manager, 2 volunteer mentors, and 2 project team members) had an average of 111 posts per month (Figure 2B). There was a moderate direct correlation (correlation coefficient: 0.59) between the number of member posts and moderator posts. On average, 19 members posted comments to the network each month (Figure 2C).

As shown in Figure 2A-C, there was a significant decline in member and moderator posts, as well as in the numbers of people posting between April 2022 and October 2022, which corresponded to a refocused effort by the community manager to define enrollment and engagement [16] strategies and targets for the network. New targets were identified in November 2022, which were approved by institutional leaders and were in place through June 2023.

During this study, 34% (n=86) of members posted on the network. Twenty percent (n=50) posted 1 to 10 times, 6% (n=14) posted 11 to 25 times, 6% (n=15) posted 26 to 100 times, and 3% (n=7) posted more than 100 times. Among those posting, the median number of posts was 6 (IQR 2‐26.5).

In total, 187 discussion topics were created, including 42% (n=78) started by members and 58% (n=109) started by moderators. Seventy-eight discussion topics each had 10 or more posts associated with them, while 32 discussion topics had only 1 post, including announcements of in-person or web-based support sessions (n=22), caregiving resources (n=4), or general network announcements (n=4).

The most popular discussion topics and respective number of posts included: “Check-in: How are we all doing today?” (n=1753), “Sources of joy and hope” (n=1436), “Can you name your feeling today? (n=649), “Meet fellow members: come introduce yourself” (n=409), “How has your grief changed over time?” (n=237), “Alzheimer Disease and other dementias - come share” (n=231), “In person meet-ups, anyone?” (n=139), “What do you wish you had known about being a care partner?” (n=109), and “What I thought I would never be thankful for...” (n=108).

#### Experience: Satisfaction and Ability to Find Meaning

**Figure 3.**
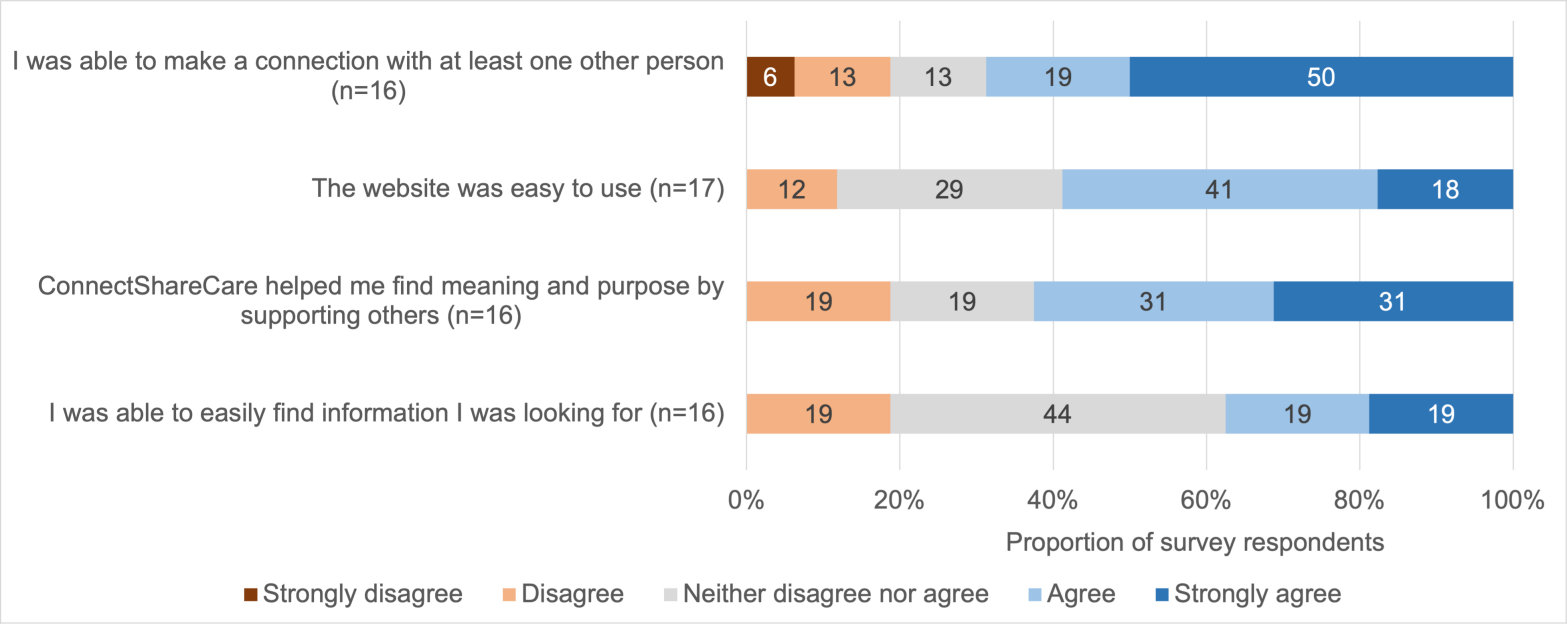
ConnectShareCare usability, as reported by ConnectShareCare members who responded to the survey about the user experience of the network during this study. Respondents with missing data were excluded from the analysis.

Care partner experience was assessed via an anonymous survey. Among those seeking support (n=16), most were satisfied with the support they received (n=12, 75%; data not shown). As shown in Figure 3, more than half of the survey respondents seeking support were able to make a connection with at least one other person (n=11, 69%) and believed that ConnectShareCare helped them find meaning and purpose by supporting others (n=10, 62%).

Among those seeking information (n=16)**,** most were satisfied with the information they found (n=14, 88%). Nonetheless, less than half reported being able to easily find information (n=6, 38%) and only 59% (n=10) found the website easy to use.

#### Value and Opportunities for Improvement

Survey respondents most commonly identified peer connection and support as the greatest value of ConnectShareCare. As expressed by a woman aged 66 years actively caring for a loved one: “I was feeling very down and, in response to a post, received ‘hug’ and ‘like’ symbols as well as words of validation and encouragement which helped me turn around my day.” A woman aged 53 years who recently lost a loved one shared the value in: “Feeling connected, talking to people who understand what I might be going through, kindred spirits.” Similarly, a woman aged 51 years who was recently bereaved identified value in: “Getting my feelings out and connecting with people. I think it is important to share stories.” Another woman aged 61 years shared the value as: “The honesty, gratitude, sharing, support that people give of themselves, and offer to others.” Respondents also noted the responsiveness of the network, as shared by this woman aged 66 years caring for a loved one: “I appreciate the caring comments from members to each other and to me. When I ask a question, I get a reply fairly quickly. I appreciate offering support to others.”

Improvement opportunities highlighted both a desire for greater engagement in the network and improved ability to access information or resources. As noted by this bereaved man (age not listed): “Continue to reach out to our regional community. As more individuals and organizations learn about CSC [ConnectShareCare] the more they will add to the CSC [ConnectShareCare] community.” Similarly, a woman aged 56 years who recently lost a loved one shared: “Not sure how you can engage more members to post? Not everyone wants to, and that’s ok. I see you trying!” Improved access to information was highlighted by a woman aged 75 years actively caring for a loved one who stated: “Maybe a little information on how to access things within the group.” The need for additional resources was highlighted by a bereaved woman aged 51 years who commented: “I think we need resources for younger people.”

## Discussion

### Principal Results

Web-based peer support networks can offer a valuable community for individuals who may not know one another, but who share common experiences. Our study found that it was feasible to enroll and engage care partners of people with serious illness and those in bereavement using a web-based peer support network. Over one-third of ConnectShareCare members posted to the digital network. Respondents reported being able to connect with others and find meaning and purpose through supporting others. While over half indicated the platform was easy to use, less than half found it easy to find the information they were seeking. Overall, the platform offered a “safe” space for people to share.

This study offered real-world testing and implementation of a web-based peer support network in a rural region with a robust moderation structure, including a community manager, volunteer mentors, and project team members. Members were welcomed, connected to others with shared lived experience, offered forum topics and posts to encourage engagement, and monitored to ensure the community rules were respected. An inherent advantage to this support group was the regional nature of the model (as opposed to a national support group), which fostered shared community knowledge, experience, and local opportunities.

Enrollment and engagement levels mirrored the effort put into them. For example, initial enrollment efforts were strong, and in response, the number of enrolled members per month was higher than when community management strategies shifted to focus on member engagement strategies. Engagement of members, as shown by posts per month and members posting per month, also reflected the focus on engagement strategies deployed by the moderators. When the number of posts from the designated community manager declined, the number of member posts decreased. The relationship between the number of moderator posts and member posts highlights the critical role of the moderator in the network, as others have found [19].

### Limitations

Our platform and study focused on those who enrolled and engaged. Participation in ConnectShareCare was open to the public, and verification as a care partner was not required, thus, members include not only care partners but also other community members or interested staff and clinicians. Enrollment and usage data reflect real-world implementation data, and patterns of use may differ if members were restricted to care partners only. Future efforts could target and engage those who may be most lonely and may benefit the most from remote interaction (eg, individuals who are homebound). Prior research has indicated a relationship between being homebound and social isolation and loneliness [20,21] and that people with these characteristics are also most likely to engage in web-based peer support communities [22-24]. In this study, all users spoke English as a primary language, and most had college degrees, which may not be representative of the general population or those who may benefit the most from a web-based peer support network. Moreover, our study did not collect racial and ethnic information on network members or survey respondents, and cultural differences may impact enrollment and engagement behaviors. Finally, survey participants ranged in age from 42 to 86 years; however, due to the small sample size, we are unable to assess potential age-related barriers or facilitators of engagement.

As this study required access to the internet and literacy, inherent selection bias existed. Due to the self-enrolling aspect of ConnectShareCare, we lack perspectives of the population of care partners who opted not to participate. Additionally, most of the completed survey responses were received from “engaged” members, and not from those who did not log in at least once in the prior 90 days. While low survey responses are common in anonymous web-based surveys [25], the limited response from those who did not engage likely overinflated positive responses regarding experiences with the network.

### Comparison With Prior Work

Members of ConnectShareCare most frequently engaged with fellow community members on discussion topics that offered a place to “check-in” and share how one was doing and feeling at a given time, as well as identifying sources of hope and joy. Other similar networks have found that discussion topics around emotions and sentiments are common among care partners of people with serious illness [26,27].

Like other web-based peer support communities [26,28-30], engagement by members of ConnectShareCare was variable, with the largest proportion (65%) not posting to the network, 32% contributing 1 to 100 posts, and a small proportion (3%) contributing more than 100 posts. While this exceeds the often referenced 90-9-1 rule for “lurkers,” “contributors,” and “super users” of a social network, studies have shown that nonposting members can gain benefits from being “part of” a web-based peer support community [30]. Recent studies have shown that nonposting members exhibit a higher level of perceived functional well-being [28] and demonstrate similar improvements as posters for being better informed, more confident in the relationship with their physician, having improved acceptance of the disease, and feeling more confident about treatment [29].

This study identified some challenges with web-based peer support communities. For example, participation was largely dominated by a small number of individuals. The ConnectShareCare network offered a variety of resources for its members; nonetheless, some survey respondents still struggled to easily find information they were seeking, which may reflect the often overwhelming nature of caring for a loved one [31]. Previous research has similarly found that engagement in web-based peer support networks is difficult for those with limited time and digital information-seeking skills [32]. This observation emphasizes an important role for the community manager to link individuals to specific resources and to optimize members’ abilities to find information within the network’s resources or through the ConnectShareCare community. Notably, following the completion of this evaluation, several enhancements were made to the platform supporting efforts to improve retention, increase findability (eg, search functions), and reduce moderator burden.

The ConnectShareCare network was co-designed to support both active and bereaved care partners, recognizing that while active and bereaved care partners may have different needs, they may benefit from connecting with each other to share information and provide or receive support [14]. Research suggests that care partners may have varying levels of interaction with web-based peer support networks due to factors such as available downtime or emotional isolation [24]. As such, differences in needs and experiences of active and bereaved care partners may impact enrollment, engagement, and care partner comfort within the network. Separate forums for active and bereaved care partners provided the ability for members to seamlessly transition between forums.

### Conclusions

This study enhanced our understanding of growth in membership enrollment, engagement, and experiences of using a web-based peer support network, ConnectShareCare, among people in a rural region of the northeastern United States. To maintain the integrity of this model, designated moderators who strive to actively facilitate connection are key to promoting participation in the network. Additional work is needed to understand the types of information that members have difficulty finding and to identify the impact of the network on health and well-being outcomes, such as distress and social connectivity. Future work in this area may wish to explore the impact of small incentives [33] to encourage people to engage in the network, expansion to non-English speaking populations, and the development of a set of critical success factors to support care partner well-being as well as health care operations. Larger studies with more representative populations of care partners are imperative for determining utility, sustainability, spread, and scalability.

## Supplementary material

10.2196/70206 Multimedia Appendix 1 Community management guidelines.

10.2196/70206 Multimedia Appendix 2 Anonymous user experience survey.
