# Supplementary material for: Feasibility and Usability of a Web-Based Peer Support Network for Care Partners of People With Serious Illness (ConnectShareCare): Observational Study
Source: JMIR Form Res. 2025 Jun 11;9:e70206. doi: 10.2196/70206 (PMC12175870; doi:10.2196/70206)

ConnectShareCare

Community Management Guidelines

Why We Moderate 2
How We Moderate 2
Moderation Options 2
Intentional spam or abuse 2
Posts that fail to comply with the community guidelines 2
Deactivate an account 3
ConnectShareCare Community Guidelines 3
Evaluating a potential crisis 3
Mentor Response 5
Keyword Monitoring 5
Responsibilities 6
Community Manager 6
Volunteer Mentors 6
Crisis Protocol 6
Volunteer Mentors 6
Community Manager 6
When to quarantine/remove a post 7
Appendices 8
Appendix A. ConnectShareCare Community Guidelines 8
Appendix B. Sign in page 9
Appendix C. Crisis Resources Page 10
Appendix D. Creating a CSC Report 11
Appendix E. Additional Resources and National Helplines 13
Appendix F. Quarantining a Post 15

Why We Moderate

At ConnectShareCare (CSC), we recognize the value of peer-to-peer sharing. Care partners, family, and friends know things — about themselves, their family members, treatments, resources, and choices, and they want to share what they know to help others. By sharing information about our health and well-being, we become a community of support. CSC's Community Manager and Volunteer Mentors are committed to keeping the community safe, supportive, responsive, inclusive, and helpful.

How We Moderate

CSC Volunteer Mentors monitor the messages posted by community members to check for information or dialogue that could be misleading or harmful. We also watch for anyone abusing the conditions outlined in the Community Guidelines in **Appendix A**. We do not edit or remove messages or ban members without good reason. Views expressed in this community are solely the views of the members and do not represent professional recommendations, policy, or advice of Dartmouth College (July 1 2023 Dartmouth Health).

The messages members post are displayed instantly without any delay as we don't have a screening process in place. While we make an effort to monitor most of the posts, we rely on our members (the community) to notify us about any concerning content. This approach allows us to maintain an active community that provides support whenever it's needed. The discussion topics and titles on CSC, Story feature and Resources (landing page) can be viewed by the public (without member registration), but the actual content within those posts is only accessible to registered members. This allows the community to be discovered by individuals seeking support and information. However, we strongly emphasize member safety and privacy and highly recommend refraining from sharing personal information such as phone numbers, addresses, emails, social security numbers, or any other private details. Any personal information shared in public posts will be promptly removed by the Community Manager.

<u>Moderation Options</u>

Intentional spam or abuse

All posts are removed and we "mute" the member. This means that they think they are posting to the community but only they can see their posts. They are not public. When they get no response from the community, they go away. This prevents them from simply creating a new account with a different email address and trying to spam again.

Posts that fail to comply with the community guidelines

We remove or edit the post and share what was done and why with members. We also track the number of posts per month which require an intervention by the Community Manager. Private CSC messaging is then used to further communicate with involved parties. The member may or may not be muted temporarily.

Deactivate an account

We can completely remove a member's account if they request it or if a decision has been made to remove them. This does not automatically remove their post. When deactivating an account, there are 3 options:

1. Leave all posts in place associated with their profile and username. This can be used when someone dies and their history in the community is their legacy.
2. Leave all posts in places but anonymize the author. Someone agrees that their posts leave a good legacy, but they no longer want their identity associated with the posts.
3. Remove all posts. Do this for bad actors. They are usually stopped before they have made multiple posts.

ConnectShareCare Community Guidelines

Part of customized Welcome Email Journey to new registered members to support website navigation, safe space and member engagement. This Email Journey was co-designed by a group including those who have lived with or cared for someone with serious illness, with advising from CY Advising (cyhealthcommunications@gmail.com) . The Community Guidelines are reviewed on an annual basis and updated as needed. See **Appendix A** for Community Guidelines

Asynchronous nature of ConnectShareCare communications

ConnectShareCare conversations do not always occur in real-time. On the sign-in page, there is a note at the bottom informing people to connect a doctor, family member or friend or dial 911 if they need immediate help (**Appendix B**).

These is also a Crisis Resources page that provides information on crisis hotlines that can be accessed 24 hours a day (**Appendix C**).

Evaluating a potential crisis

When assessing how and when to respond to a potential crisis, it is crucial to prioritize the well-being of the individual member involved and the community's overall well-being. Additionally, it is important to be mindful of how the public message might have a negative impact on other community members. To facilitate the evaluation process, here are some key questions to consider:

1. Is this the member's first post, or have they been actively participating in the community before? Understanding the member's history can provide valuable context for the situation at hand.
2. Is the behavior exhibited in the post out of character for the member, or is it a new development? Assessing whether the post aligns with the member's typical conduct can help determine if there are underlying factors contributing to the situation.
3. How might this post impact other community members? Recognizing the potential effects on different individuals is vital in assessing the gravity of the problem and considering appropriate response measures.

4. Have any other community members responded to the post? If there have been responses, evaluating how effectively those individuals addressed the member is essential. Positive and supportive responses indicate a healthy community dynamic, which may influence the type of intervention required. However, it is still necessary to consider potential follow-up actions.
5. Should the community be aware that community management is actively monitoring and has responded to the situation? Depending on the specific scenario, it may be beneficial to acknowledge the intervention publicly. Transparency in community management can instill a sense of reassurance and confidence among community members.

Remember, exercising sound judgment based on the particular circumstances is crucial throughout the evaluation process.

Below are some example templates that may be used to tailor a specific response:

****

Example 1

Hi @[add username]. Welcome to ConnectShareCare, a safe place where you can talk – even about the tough stuff. I'm so sorry you are going through this. I understand how difficult it can be to [reference the issues they discuss in their post]. You are not alone! I'm tagging [only add people who you know will respond quickly and appropriately] who have dealt with similar issues and may be able to provide some support. The community is here for you!

If at any point you start to feel like you may consider hurting yourself, please call the National Suicide Prevention Lifeline at 988 for immediate help. Your safety is the most important thing to us and needing to talk to a professional doesn't show weakness, it shows strength!

Are you safe right now?

Example 2

The ConnectShareCare community understands that you are in a difficult place right now and we want you to know that we are here for you. This is a safe place to talk as you feel the need. I also want you to know about the National Suicide Prevention Lifeline at 988 that you can call any time for immediate help. Please call them if you are feeling threatened by your feelings of self-harm.

Example 3 Domestic hotline

If someone talks about being abused or not feeling safe, you may wish to tell them about the National Domestic Violence Hotline website http://www.thehotline.org/

***

Hi @[add username]

You have taken the first important step on the path to safety by telling someone and posting to the community. Please visit the National Domestic Violence Hotline website http://www.thehotline.org/ On this website you will find a phone number to call 988 or you can use the online chat and get help without saying a word.

By calling the Hotline, you can work with professionals to find safety and a solution that is right for you

Mentor Response

It is the responsibility of the Community Manager to conduct all follow-up related to community safety. This is not the responsibility or expectation for Volunteer Mentors. However, Mentors do have an obligation to Report behavior so the Community Manager can be notified in a timely manner, investigate and intervene as needed.

It can be tricky to interpret the intent and meaning when someone posts a message that may indicate violence and self-harm. This is particularly important when the current end-users of CSC (as of June 2023) are active and bereaved care partners of serious illness. If a post makes anyone uneasy and unsure how to respond, Report it immediately. This is a responsibility of Volunteer Mentors and supports the Community Manager - see **Appendix D** on how to create a CSC report

Keyword Monitoring

Posts about giving up, wanting suffering to end, self-harm and explicit mentions of suicide are not uncommon in online communities where members have gained trust that they can be open and honest with their feelings (also more likely in the space of serious illness). In many (most) instances, such expressions indicate that members recognize that they are posting to a supportive community where they can talk frankly about how they are feeling (this actually indicates success). However, such posts can be unsettling for the community and for Community Managers (moderators) and Volunteer Mentors.

When these words are included in content submitted by a user, an email notification will be automatically sent to the CSC Administrative Email which is monitored daily.

Keyword Monitoring List Built into Online Vendor, CareHubs (updated June 22 2023)

Safety

- suicide
- self-harm
- took his life
- took her life
- Overdose
- End it all
- I can't go on
- I want to die
- Kill

- Attack
- Assault
- Threaten
- Beat up

Responsibilities

Community Manager

It is the responsibility of the Community Manager to deal with any explicit or non-explicit mentions of suicide, self-harm and members in crisis. The CM should respond both publicly and privately to the member for different strategic reasons. See details of **crisis protocols** below, **Appendix E**.

Volunteer Mentors

It is the responsibility of Volunteer Mentors to report any post that they feel contains the potential for self-harm or abuse to the Community Manager immediately. Volunteer Mentors may respond, but it is not a volunteer obligation. This can be done via the CSC Report functions - see **Appendix D**. In addition to utilizing the Reporting function, a direct message to the Community Manger indicating that a report had been submitted can help with expedited review. It is the Community Manager's responsibility to notify Volunteer mentors that the Report has been received (without issue) and is being investigated. A summary of action taken is also necessary when concluding follow-up. Reports and follow-up are monitored, documented on a monthly basis to maintain a safe space, support learning and improvement.

Crisis Protocol

Volunteer Mentors

Volunteer mentors may have personal and professional experience with suicide, self-harm and counselling, and can be very equipped to respond to members. Respond where you can offer support and help IF you are comfortable doing so but again this is not the expectation for this role. Hearing from someone who has been there is extremely helpful and powerful. Remember, responding is not a Volunteer Mentor/moderator obligation - reporting is a duty. The follow-up is owned by the Community Manager.

Community Manager

It can be challenging to know when to post information referring a member to the suicide hotline. Sometimes escalating to this point right away can shut down a conversation, turning away the person who is seeking support and/or sharing. Trust your instinct and contact a colleague to discuss if necessary.

When someone explicitly threatens suicide, self-harm or describes that they are in crisis, the Community Manager will Post a public reply to the member, and depending on the circumstance will:

- Ask the person via private messaging to call 9-1-1 or go to their nearest emergency room immediately as well as provide them information about the National Suicide and Crisis Hotline and to **Dial or Text 988**
- Send a private message to the member to ask for their contact information, particularly their present location. The CM may call the nearest police department and ask them to do a safety check on this individual. Be prepared to identify yourself, who you represent, the reason you are calling and the name and present location of the person in question.

When to quarantine/remove a post

Posts should not be deleted/removed, but Quarantined and left on CSC, especially when appropriate responses follow (i.e. where to get services) and a helpful conversation from members follows the initial crisis. Quarantine status is meant for discussions that are potentially divisive and require further review. They are only viewable in the Admin directory - regular users cannot see or interact with them.

Quarantine suicidal crisis that are very graphic or very obviously personal. Delete if needed.

1. Record (screengrab)
2. Remove
3. Respond – try to take conversation ‘offline’ to determine whether the person is actually at risk or just exaggerating/satirical.
4. Report as above (including in monthly safety metric)

See **Appendix F** for directions.

Appendices

Appendix A. ConnectShareCare Community Guidelines

- **Be Kind**
  - Don't impose your beliefs on others (avoid religion and politics.)
  - Ask questions to help you understand the views of others.
  - Know that using sarcasm or jokes can be taken the wrong way. Don't personally attack members or health care providers.

- **Watch your language**
  - Don't use obscene or hateful language.
  - Don't type in ALL CAPS. People may think you are shouting at them.

- **Don't give out medical advice**
  - This site is not meant to give you medical advice or recommend a course of treatment and you should not rely on it to provide you with a recommended course of treatment. It is not intended, and should not be used, to replace the advice or care provided by your healthcare professional. Before making any treatment decisions, you should consult with your healthcare professional and discuss your treatment options.

- **Don't advertise, solicit, or sell anything.**

- **Be Safe**
  - We try to protect your privacy but need your help!
  - Don't share your personal information (or anyone else's.) Don't share phone numbers, addresses or social security numbers. It is your decision whether to use your real name.
  - Keep your password confidential.

- **Follow the rules.**
  - By posting to ConnectShareCare, you agree to abide by our Terms of Use.
  - We are committed to protecting your privacy. See our Privacy Policy.
  - See our Disclaimer.

Appendix B. Sign in page

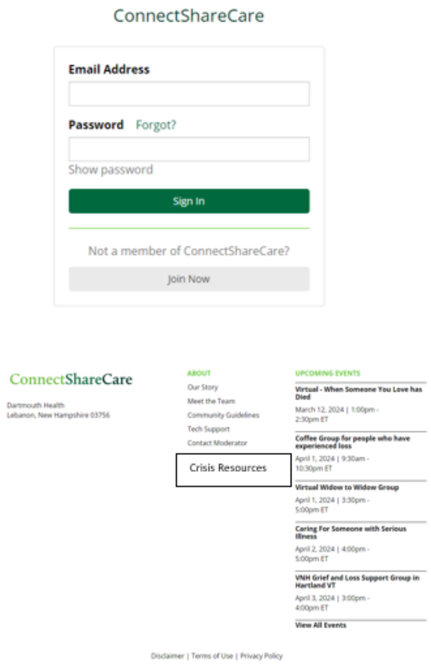

ConnectShareCare provides a space to connect with other care partners for emotional support and useful information. If you need help right away, please reach out to your health care provider, a family member or friend, or dial 911.

Appendix C. Crisis Resources Page

Crisis Resources

ConnectShareCare provides a space to connect with other care partners for emotional support and useful information. A response to your post may not happen right away. There may be times when you want to connect with someone immediately.

Below are helplines if you are in need of immediate attention. This information is not a substitute for professional advice or care. If you are in need of help, please reach out to your health care provider, one of the helplines below, a family member or friend, or dial 911.

Suicide Hotlines:
National Suicide Prevention Lifeline 988
Crisis Text Line Text HOME to 741741
US Veteran Crisis Line

Confidential Hotline for Veterans or their loved ones - 988 (press 1)
Or Confidential Veterans Chat - Text to 838255

If you are located in **Vermont**, please call your local community crisis line below or or text VT to 741741:

Addison: Counseling Service of Addison County 802-388-7641
Bennington: United Counseling Services 802-442-5491
Caledonia: NKHS 802-748-3181
Chittenden: Howard Center 802-488-7777
Essex: NKHS 802-334-6744
Franklin and Grand Isle: Northwestern Counseling and Support 802-524-6554
Lamoille: Lamoille County Mental Health 802-888-5026 on weekdays 8AM-4:30PM and 802-888-8888 on nights and weekends
Orange: Clara Martin Center 1-800-639-6360
Orleans: NKHS 802-334-6744
Rutland: Rutland MH Services 802-775-1000
Washington: Washington County MH Services, 802-229-0591
Windham: HCRS 1-800-622-4235
Windsor: HCRS 1-800-622-4235

For further information for VT residents: https://mentalhealth.vermont.gov/services/emergency-services/how-get-help

Appendix D. Creating a CSC Report

1. **Click on the three dots to the right of the post of concern.**

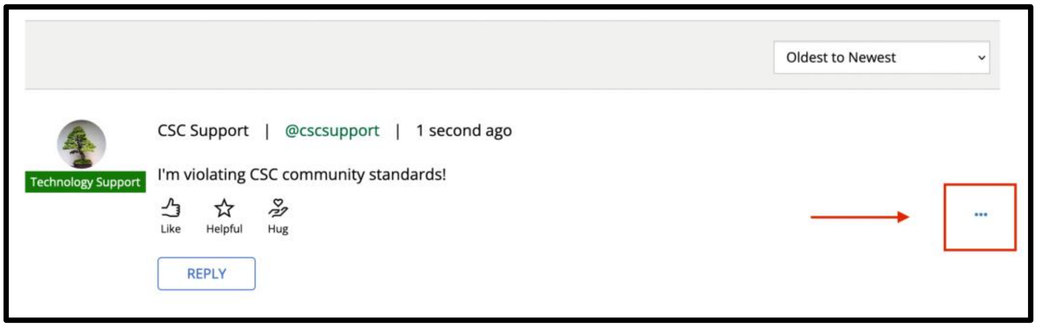

2. **Click on "report this comment"**

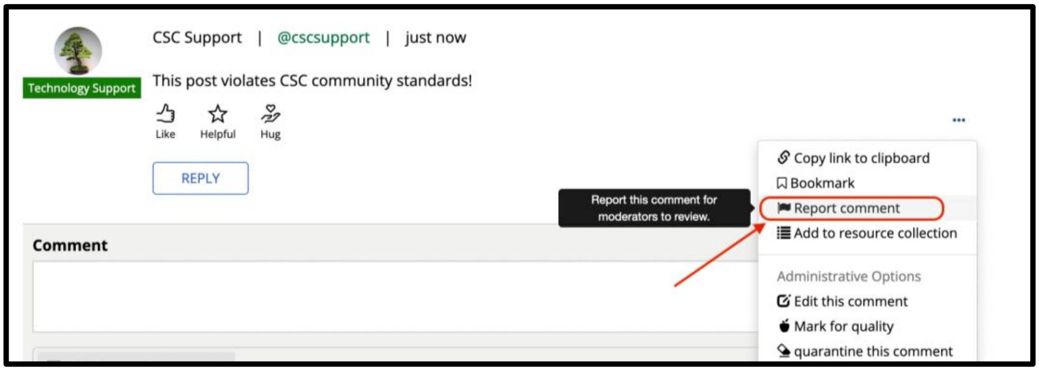

3. **Provide a brief description as to the reason you are reporting the post**
4. **Click on "submit"**

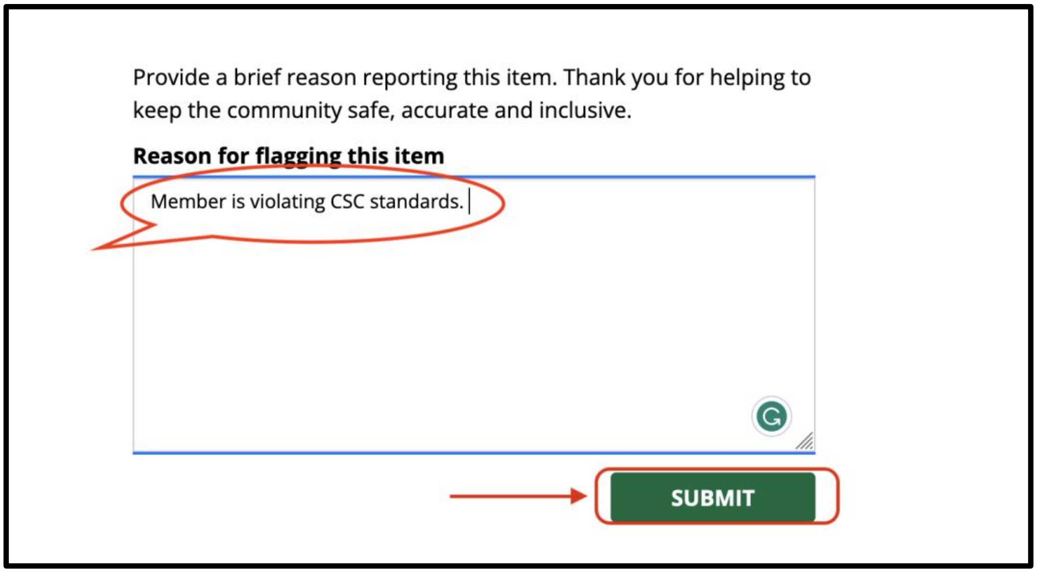

Note: Admins on ConnectShareCare have access to all reports - https://connectsharecare.org/admin/manage/social/reported/

Appendix E. Additional Resources and National Helplines

Suicide Hotlines

- American Association of Suicidology http://www.suicidology.org/
- About the Suicide Prevention Lifeline http://www.suicidepreventionlifeline.org/gethelp/someone.aspx
- We can all prevent suicide http://www.suicidepreventionlifeline.org/learn/warningsigns.aspx
    - National Suicide Prevention Lifeline 988
- Crisis Text Line Text HOME to 741741
- US Veteran Crisis Line
    - Confidential Hotline for Veterans or their loved ones - 988 (press 1)
    - Or Confidential Veterans Chat - Text to 838255

Addiction Hotlines

- Alcoholics Anonymous: (212) 870-3400
- Narcotics Anonymous: (818) 773-9999
- Al-Anon/Alateen Family Group Services: (800) 356-9996 or (888) 4AL-ANON
- National Association for Children of Alcoholics: (888) 554-COAS
- National Drug Information Treatment and Referral Hotline: (800) 662-HELP (4357)
- National Organization of Students Against Substance Abuse

Crisis counseling

- Covenant House (800) RUNAWAY

  Crisis Care for Homeless and At-Risk Kids, state specific resources available through their website
- National Mental Health Association: (800) 969-6642 9AM-5PM Mon-Fri

  Information on mental health topics and referrals, access to an info specialist
- National Youth Crisis Hotline: (800) 442-HOPE (4673)

  Provides counseling and referrals to local drug treatment centers, shelters, and counseling services. Responds to youth dealing with pregnancy, molestation, suicide, and child abuse. Operates 24 hours, seven days a week.
- Crisis Text Line Text HOME to 74174

Abuse & Neglect

- National Domestic Violence Hotline (800) 799-SAFE or (800) 787-3224 (TDD)

- Rape, Abuse and Incest National Network (800) 656-HOPE (4673)
- Child Abuse Hotline (800) 422-4453
- National Center for Missing & Exploited Children (800) THE-LOST (843-5678)
- Child Find of America Hotline (800) I-AM-LOST (426-5678)
- Resources by state for Elder Abuse Hotlines
- Resources by state for Adult Protective Services Hotlines
- Self-Abuse (800) DONTCUT

Poison Control

- American Association of Poison Control Centers (800) -222-1222

Appendix F. Quarantining a Post

1. **Click on the three dots to the right of the post of concern.**

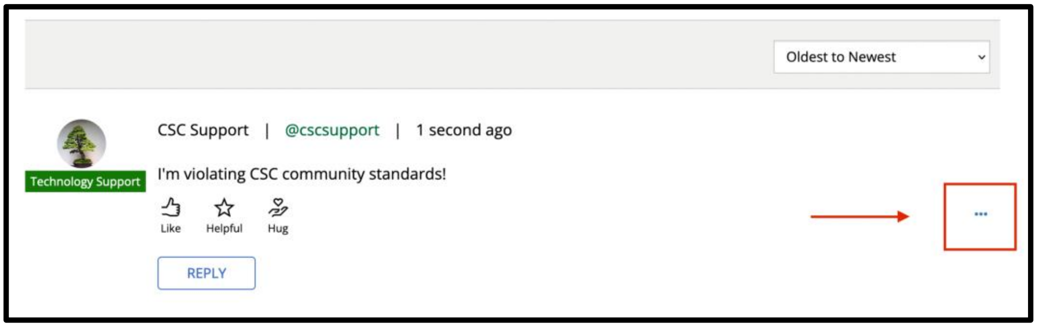

2. **Click on “quarantine this comment”**

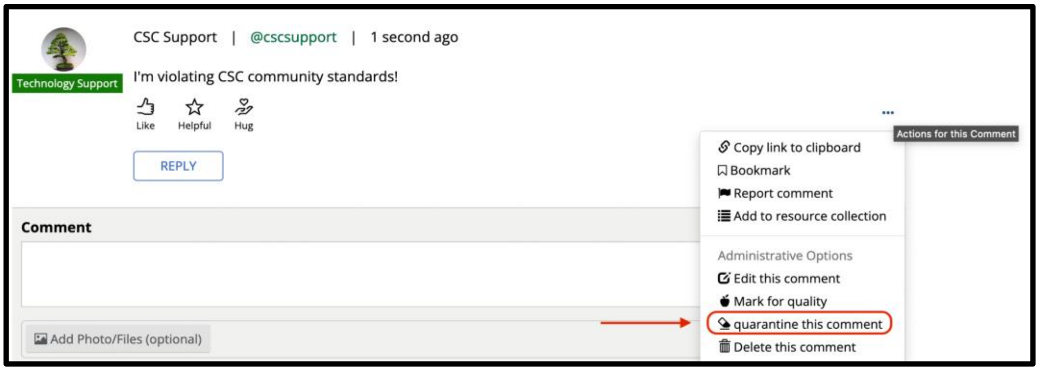

3. **Click on “ok” to confirm quarantining the comment**

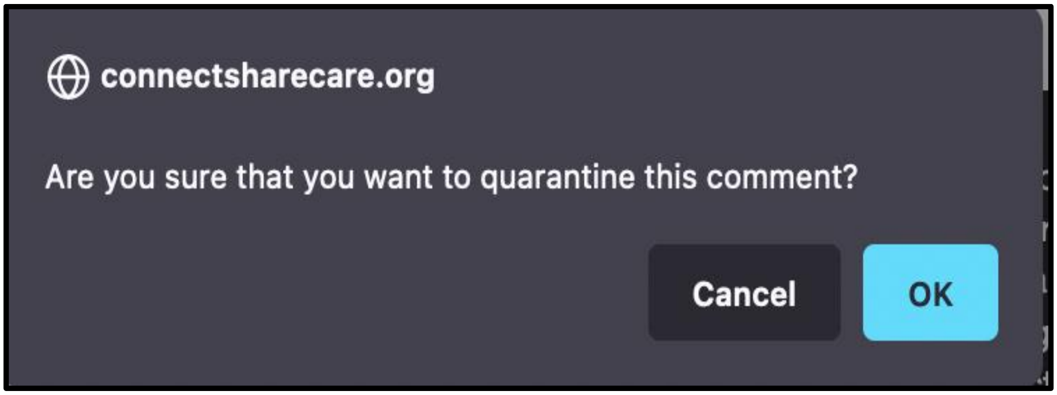

Supplement: Multimedia Appendix 1 [file formative-v9-e70206-s001.pdf]
