# Supplementary material for: Feasibility and Usability of a Web-Based Peer Support Network for Care Partners of People With Serious Illness (ConnectShareCare): Observational Study
Source: JMIR Form Res. 2025 Jun 11;9:e70206. doi: 10.2196/70206 (PMC12175870; doi:10.2196/70206)
Supplement: Multimedia Appendix 2 [file formative-v9-e70206-s002.docx]

**ConnectShareCare Anonymous User Experience Survey**

Thank you for being part of the ConnectShareCare.org community, an online network designed to help active and bereaved care partners and caregivers of persons with a serious illness connect through lived experiences. The Dartmouth Institute for Health Policy and Clinical Practice (TDI) is seeking your feedback about your experience using ConnectShareCare.org. Your feedback is very important to us in creating a user-friendly and meaningful support network that is helpful to you and others.

Your participation is voluntary and will have no impact on your ability to access and use ConnectShareCare.org. You may choose to not answer any or all of the questions. The information collected will be maintained confidentially by the TDI research team. No identifying information will be collected or used in any presentation or publications about this project. Your feedback will be used to improve ConnectShareCare.org

**The following questions ask about your experience with ConnectShareCare.**

1. How long have you been a member of ConnectShareCare?

1-3 months 4-6 months 7-12 months More than 12 months

2. How did you hear about ConnectShareCare?

Dartmouth-Hitchcock Health clinician or staff

Listserv/Social Media Post

Community Organization (ex. ServiceLink, Bayada Hospice and Palliative Care)

Friend, family member, or neighbor

Flyer or pamphlet

Other _________________________

3. Which features of ConnectShareCare do you most frequently use? (Check all that apply)

Support Groups - *Just Want to Talk*

Support Groups - *How to Use ConnectShareCare*

Private messaging to other ConnectShareCare members

Events Calendar

Resources (categories such as Planning Ahead, Practical, Emotional, Communication, or Family Resources)

Stories

Other __________________________________________________

4. How often do you visit ConnectShareCare?

Daily Weekly Monthly Quarterly Never Other ___________

5. At what time of day to you prefer to access ConnectShareCare?

10pm to 2am 2am to 7am 8am to 5pm 5pm to 10pm

6. What would make it more likely for you to visit ConnectShareCare?

________________________________________________________________

7. What do you like most about the ConnectShareCare support network?

________________________________________________________________

8. Please answer the following questions based on your use of ConnectShareCare over the past month.

|  | Strongly disagree | Disagree | Neither agree nor disagree | Agree | Strongly agree |
| --- | --- | --- | --- | --- | --- |
| The website was easy to use. |  |  |  |  |  |
| I was able to easily find information I was looking for. |  |  |  |  |  |
| I was able to make a connection with at least one other person. |  |  |  |  |  |
| ConnectShareCare helped me find meaning and purpose by supporting others. |  |  |  |  |  |

9. Overall, how satisfied are you with the support you received through ConnectShareCare?

Very satisfied Somewhat satisfied Neither satisfied nor dissatisfied

Somewhat dissatisfied Very dissatisfied Not applicable. I was not seeking support.

10. What could we do to improve the support available through ConnectShareCare?

________________________________________________________________

11. Would you prefer to see separate discussion sections for those who are currently providing care/support to someone with a serious illness and those who have lost a loved one to a serious illness?

Yes, separate groups No, combine groups Unsure Other __________

12. Overall, how satisfied are you with the information you found through ConnectShareCare?

Very satisfied Somewhat satisfied Neither satisfied nor dissatisfied

Somewhat dissatisfied Very dissatisfied Not applicable, I was not seeking information or resources.

13. What could we do to improve the information and resources you found through ConnectShareCare?

________________________________________________________________

14. Are there additional resources or supports that you would like to see as part of ConnectShareCare?

________________________________________________________________

15. Optional: Please share an example of a time where ConnectShareCare made a positive impact on your day, or on your ability to connect with others or manage daily activities?

________________________________________________________________

16. NPS On a scale from 0-10, how likely are you to recommend ConnectShareCare to another care partner of a person with serious illness, or a person in bereavement?

0 1 2 3 4 5 6 7 8 9 10

17. What changes could we make to ConnectShareCare for you to give it a higher rating?

________________________________________________________________

**The following questions ask about your role in caring for someone with a serious illness, your well-being, and how ConnectShareCare can help support you.**

18. Which of the following best describes you? (Check all that apply)

I currently care for/provide support to someone with a serious illness

I have lost a loved one to a serious illness

Other __________________________________________________

19. What is your relationship to the person for whom you care or cared for?

Child Friend/Neighbor Grandchild Parent Sibling (Brother/Sister)

Spouse/Life Partner Other __________________________________________________

20. Would you consider yourself to be, or have been, the primary care partner?
 ** A primary caregiver is the person responsible with coordination of care of a friend or loved who requires assistance in some or all components of their care.*

Yes No

21. What is the primary diagnosis of the person for whom you are or were providing care or support?

________________________________________________________________

22. How long have you been caring for/providing support to someone with a serious illness? *[Currently caring for someone with a serious illness]*

Less than 1 month 1-3 months 4-6 months

7-12 months 1-2 years More than 2 years

23. How much time has passed since the death of the person for whom you were providing care or support? *[Lost a loved one to serious illness]*

Less than 1 month 1-3 months 4-6 months

7-12 months 1-2 years More than 2 years

**The following questions ask about your ability to connect with others, either within or outside of ConnectShareCare.**

24. Please respond to each item by marking one box per row.

|  | Never | Rarely | Sometimes | Often | Always |
| --- | --- | --- | --- | --- | --- |
| I have someone who will listen to me when I need to talk. |  |  |  |  |  |
| I have someone to confide in or talk to about myself or my problems. |  |  |  |  |  |
| I have someone who makes me feel appreciated. |  |  |  |  |  |
| I have someone to talk with when I have a bad day. |  |  |  |  |  |

25. Please respond to each item by marking one box per row.

|  | Never | Rarely | Sometimes | Often | Always |
| --- | --- | --- | --- | --- | --- |
| I feel left out. |  |  |  |  |  |
| I feel that people barely know me. |  |  |  |  |  |
| I feel isolated from others. |  |  |  |  |  |
| I feel that people are around me but not with me. |  |  |  |  |  |

26. Have you experienced any changes in your ability to connect with others since joining ConnectShareCare?

Much improved Somewhat improved No change Somewhat worse Much worse

27. In the last seven days... In general, how would you rate your satisfaction with your social activities and relationships?

Excellent Very good Good Fair Poor

28. Have you experienced any changes in your satisfaction with your social activities and relationships since joining ConnectShareCare?

Much improved Somewhat improved No change Somewhat worse Much worse

29. What, if anything, can ConnectShareCare do to improve your ability to connect with others?

________________________________________________________________

30. Distress is an unpleasant experience of a mental, physical, social, or spiritual nature. It can affect the way you think, feel, or act. Please select the number (0-10) that best describes how much distress you have been experiencing in the past week including today. 0 = no distress and 10 = extreme distress.

0 1 2 3 4 5 6 7 8 9 10

31. What is the primary source of your distress?

________________________________________________________________

32. Have you experienced any changes in distress since joining ConnectShareCare?

Much improved Somewhat improved No change Somewhat worse Much worse

33. What, if anything, can ConnectShareCare offer to help lower your distress?

________________________________________________________________

**The following questions ask about you.**

34. What is your gender?

Male Female Prefer to self-describe ______________ Prefer not to answer

35. Age What is your age (in years)?

____________________

36. What is your primary language?

English French Spanish Other ________________________________

37. What is your highest level of education?

Less than high school High school diploma or equivalent Some college

College graduate Master's/doctoral level degree Unknown/not applicable

38. Employment Are you currently a...? (Check all that apply)

Full time care partner/caregiver Full time employee Part time employee

Military Retired Out of work and looking for work Other __________________
